# Immune Checkpoint Inhibitors with or without Radiotherapy in Non-Small Cell Lung Cancer Patients with Brain Metastases: A Systematic Review and Meta-Analysis

**DOI:** 10.3390/diagnostics10121098

**Published:** 2020-12-16

**Authors:** Dong Yeong Kim, Pyeong Hwa Kim, Chong Hyun Suh, Kyung Won Kim, Ho Sung Kim

**Affiliations:** 1 Department of Quarantine, Incheon Airport National Quarantine Station, Incheon 22382, Korea; keo8410@gmail.com; 2 Department of Radiology and Research Institute of Radiology, Asan Medical Center, University of Ulsan College of Medicine, Seoul 05505, Korea; phkim@amc.seoul.kr (P.H.K.); medimash@gmail.com (K.W.K.); radhskim@gmail.com (H.S.K.)

**Keywords:** non-small cell lung cancer, immune checkpoint inhibitors, intracranial response, meta-analysis

## Abstract

This study aimed to evaluate the radiologic response and adverse event rates of immune checkpoint inhibitor (ICI) therapy with or without radiotherapy for the treatment of non-small cell lung cancer (NSCLC) brain metastases. A systematic literature search was performed up to January 3, 2020. Studies evaluating the intracranial objective response rates (ORR) and/or disease control rates (DCR) of ICI with or without radiotherapy for treating NSCLC brain metastases were included. Consequently, twelve studies satisfied inclusion criteria. ICI combined with radiotherapy (pooled ORR, 95%; DCR, 97%) showed better local efficacy compared to ICI monotherapy (pooled ORR, 24%; DCR, 44%; *p* < 0.01 for both ORR and DCR). Grade 3 or 4 central nervous system (CNS)-related adverse event rates were not different (5% vs. 4%; *p* = 0.93). In conclusion, ICI combined with radiotherapy showed better intracranial efficacy than ICI monotherapy for treating NSCLC brain metastases. CNS-related grade 3 or 4 adverse event rate was not statistically different between the two groups. Several prospective trials are needed to compare the efficacy of ICI combined with radiotherapy and ICI monotherapy.

## 1. Introduction

The brain is a common site of metastases in patients with advanced non-small cell lung cancer (NSCLC). Approximately 10–15% of NSCLC patients get detected with brain metastasis at the initial diagnosis [1], and 24–44% experience brain metastasis at some point during their medical treatment [2]. Multiple lesions are common in brain metastasis, and in case of NSCLC, it is observed in approximately half of the patients [3]. The prognosis is dismal, with median overall survival being approximately 7 months [4].

Immunotherapy is now a standard therapy for the patients with advanced NSCLC with PD-L1 expression based on multiple clinical trial results [5,6]. However, patients with brain metastases were mostly excluded from the pivotal trials using immunotherapy, and the efficacy of immunotherapy in those patients were not fully evaluated. Currently, the whole brain or stereotactic radiation therapy and surgery are the mainstay of treatment for the brain metastases. Recent studies showed promising results of immunotherapy with or without combined radiation therapy for the brain metastasis, with objective response rate (ORR) of 9–40% [7,8,9,10,11,12] using ICI therapy alone, and 95–100% with combination of ICI therapy and radiotherapy [13,14]. In one study, patients with NSCLC who were treated with pembrolizumab and who received previous radiation therapy showed improved therapeutic response [15]. These results suggest a synergistic effect between radiation and ICI. However, their data were not sufficient to compare differences in therapeutic response between the available treatment arms (i.e., ICI monotherapy, ICI combined with chemotherapy, ICI combined with radiotherapy). Therefore, the purpose of our meta-analysis was to evaluate the local efficacy and safety of different treatment options using ICI for the treatment of NSCLC brain metastases.

## 2. Materials and Methods

This review was conducted according to the Preferred Reporting Items for Systematic Reviews and Meta-Analyses (PRISMA) guidelines [16].

### 2.1. Search Strategy and Eligibility Criteria

A computerized search of the MEDLINE/PubMed and EMBASE databases was performed using Medical Subject Headings (MeSH) or EMTREE terms to find relevant articles until 3 January 2020. The search keywords were as follows: ((lung cancer) or (non small cell lung cancer) or (NSCLC)) and (brain metasta*)) and ((CTLA4) or (CTLA-4) or (PD1) or (PD-1) or (PD-L1) or (ipilimumab) or (nivolumab) or (pembrolizumab) or (atezolizumab) or (avelumab) or (durvalumab)). The search was not limited by any filters.

After eliminating identical articles, we screened the articles by the titles and abstracts for relevance. Full-texts were evaluated depending on the following eligibility criteria: (a) patients: NSCLC patients with brain metastases; (b) intervention: ICI with or without radiotherapy; (c) comparator(s)/control: not applicable; (d) outcomes: intracranial ORR or disease control rate (DCR); and (e) study design: observational studies and clinical trials published as original articles. Studies were excluded if any of the following criteria were met: (a) other types of publications including conference abstracts, reviews, case reports, comments, editorials, and letters and (b) studies providing insufficient information for calculating the intracranial results of the intervention.

### 2.2. Data Extraction and Quality Assessment

Using a standardized extraction form we obtained the following data on study design and results: (a) study characteristics: country and institution of origin, recruitment period, study design (retrospective vs. prospective); (b) demographic and clinical characteristics: number of treated patients/lesions, presence vs. absence of symptoms associated with NSCLC brain metastasis; (c) intervention characteristics: treatment arms (ICI combined with chemotherapy vs. ICI combined with radiotherapy vs. ICI monotherapy), ICI used (e.g., atezolizumab, pembrolizumab, and nivolumab); and (d) characteristics account for outcomes: response assessment criteria, response assessment time after the first therapy. We evaluated the quality of the evidence in the included studies using the Grading of Recommendations Assessment, Development, and Evaluation (GRADE) system [17,18]. The GRADE system ranks the quality of evidence from very low to high based on study design, risk of bias, imprecision, inconsistency, indirectness, the magnitude of the effect, dose-response relationship, and consideration of all plausible residual confounders.

### 2.3. Data Synthesis and Analysis

The primary outcomes of this meta-analysis are (a) intracranial ORR (percentage of patients with NSCLC brain metastases who confirmed complete [CR] or partial response [PR]) and (b) intracranial DCR (percentage of patients with NSCLC brain metastases who confirmed CR, PR, or stable disease [SD]) evaluated with the response assessment criteria of each included study. Results were pooled according to the treatment arms (ICI monotherapy vs. ICI combined with radiotherapy vs. ICI combined with chemotherapy). In addition, the intracranial CR rate was also meta-analytically pooled.

We also assessed the safety-associated outcomes, including treatment-related grade 3 or 4 adverse events and CNS-related grade 3 or 4 adverse events, following the Common Terminology Criteria for Adverse Events (CTCAE) v3.0 or 4.0, and according to each selected prespecified study.

Meta-analytic pooling was based on the inverse variance weighting method to calculate weights, and the clinical response and grade 3 or 4 adverse event rates with their 95% confidence intervals (CI) were meta-analytically pooled using DerSimonian–Laird (random-effects modeling) method and fixed-effects modeling, respectively. Heterogeneity was evaluated using the following tests: (a) Cochran’s Q test, with *p* < 0.05 indicating the presence of heterogeneity, and (b) Higgins inconsistency index (*I*^2^), with *I*^2^ > 50% indicating the presence of heterogeneity [19,20,21]. To test whether treatment arms as moderators have statistical effects on meta-regression, we used Wald-type chi-square tests with multiplicity adjustment and the regression coefficient obtained was used to estimate the intervention effect from a reference group [22,23]. Statistical analyses were conducted using R software (version 3.6.1.; R Foundation for Statistical Computing, Vienna, Austria) with the “meta” and the “metafor” packages.

## 3. Results

### 3.1. Literature Search

**Figure 1:**
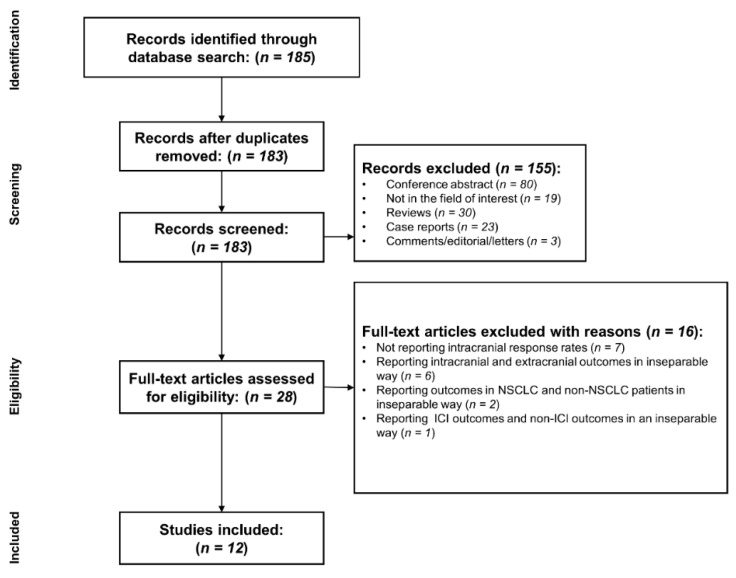
Flowchart of the study selection process.

A flow chart describing the study selection process is presented in Figure 1. After excluding two duplicates, a total of 183 studies were identified. Of these, 155 articles were excluded according to their titles and abstracts for the following reasons: (a) conference abstract (*n* = 80); (b) reviews (*n* = 30); (c) case reports (*n* = 23); (d) not in the field of interest (*n* = 19); and (e) comments/editorial/letters (*n* = 3). On the basis of the eligibility criteria, a full-text review of 28 potentially eligible studies was performed. A further exclusion of 16 articles was made according to the following reasons: (a) not reporting intracranial response rates (*n* = 7); (b) reporting intracranial and extracranial outcomes in an inseparable way (*n* = 6); (c) reporting outcomes in NSCLC and non-NSCLC patients in an inseparable way (*n* = 2); and (d) reporting ICI outcomes and non-ICI outcomes in an inseparable way (*n* = 1). Finally, a total of 12 studies (separated according to the treatment arms; seven cohorts treated with ICI monotherapy; one cohort treated with ICI combined with chemotherapy; four cohorts treated with ICI combined with radiotherapy) were included in our study [7,8,9,10,11,12,13,14,24,25,26,27].

### 3.2. Characteristics of the Included Studies

Detailed study characteristics are described in Table 1. Four studies were multicenter studies [8,9,11,24], one study was a phase II clinical trial [10], and the remaining studies were retrospective. Response Evaluation Criteria in Solid Tumors (RECIST) v1.1, Modified RECIST v1.1, Response Assessment in Neuro-Oncology-brain metastases (RANO-BM), and immunotherapy RANO (iRANO) were applied as tumor response criteria for four [7,9,12,25], three [8,10,11], two [13,14], and one [26] studies, respectively. Two studies did not report their response criteria [24,27]. Response assessment time after first therapy ranged from 1.5–3 months. Five studies did not mention their response assessment time [8,11,12,25,27]. Seven studies used ICI monotherapy [7,8,9,10,11,12,24], one study used both ICI and chemotherapy [25], and four studies used both ICI and stereotactic radiosurgery (SRS) [13,14,26,27]. Two studies recruited only asymptomatic NSCLC brain metastasis patients [8,10]. Nine studies used per-patient analysis [7,8,9,10,11,12,13,24,25], and the remaining three studies used per-lesion analysis [14,26,27].

### 3.3. Quality Assessment

Detailed quality assessments are described in Table 2. The phase II clinical trial by Goldberg et al. was initially rated with a high certainty rate [10], and the other eleven retrospective studies were initially rated with a low certainty rate [7,8,9,11,12,13,14,24,25,26,27]. In the risk of bias domain, three studies were down-rated as they performed per-lesion analysis [14,26,27]. In the imprecision domain, four studies were down-rated because of the wide 95% CI for the local efficacy, derived from a small sample size of 5 [8,12,24,25]. In the inconsistency domain, the study by Kim et al. was down-rated because of a discrepancy of local efficacy compared to the other studies using ICI monotherapy [11]. In the indirectness domain, the study by Hendriks et al. was down-rated because it only included patients who presented leptomeningeal seeding [24]. The study by Singh et al. was up-rated due to a large effect size (comprising 82% [291 out of 356 patients/lesions] among the studies including ICI combined with radiotherapy) [14]. Consequently, the quality of evidence was high in one study [10] low in four [7,9,13,14], and very low in seven studies [8,11,12,24,25,26,27].

### 3.4. Efficacy

The pooled intracranial ORR and DCR are described in Table 3. Six [7,8,9,10,11,12], one [25], and two [13,14] articles reported on intracranial ORR for ICI monotherapy, ICI combined with chemotherapy, and ICI combined with radiotherapy, respectively.

**Figure 2:**
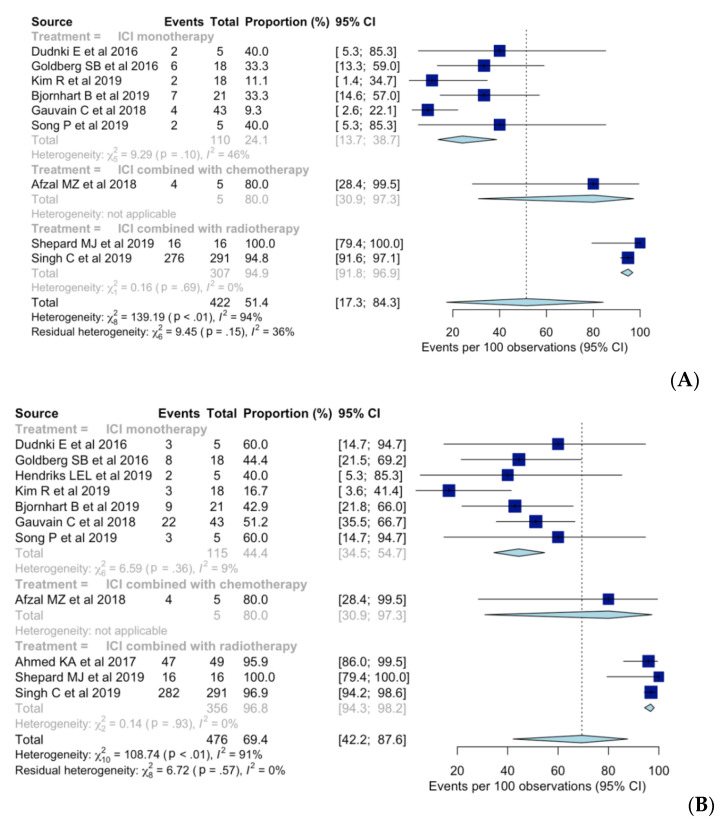
Forest plot of the intracranial (**A**) objective response rates and (**B**) disease control rates when using immune checkpoint inhibitor (ICI) monotherapy, ICI combined with chemotherapy, and ICI combined with radiotherapy.

Overall intracranial ORR was 51% (95% CI, 17–84%), with a substantial heterogeneity (*I*^2^ = 94%; *p* < 0.01) (Figure 2). Pooled intracranial ORR with random-effects modeling was 24% (95% CI, 14–39%), 80% (95% CI, 31–97%), and 95% (95% CI, 92–97%) for ICI monotherapy, ICI combined with chemotherapy, and ICI combined with radiotherapy, respectively. No substantial heterogeneity was found in any of the three subgroups. Compared to ICI monotherapy, intracranial ORR was significantly higher for ICI combined with radiotherapy (OR [95% CI], 2.32 [1.96–2.75]; *p* < 0.01), while no significant difference was found for ICI combined with chemotherapy (OR [95% CI], 1.90 [0.76–4.79]; *p* = 0.14).

Seven [7,8,9,10,11,12,24], one [25], and three studies [13,14,26] reported intracranial DCR when using ICI monotherapy, ICI combined with chemotherapy, and ICI combined with radiotherapy, respectively. Overall intracranial DCR was 69% (95% CI, 42–88%), with a substantial heterogeneity (*I*^2^ = 91%; *p* < 0.01) (Figure 2). When using ICI monotherapy, based on random-effects modeling, pooled intracranial DCR was 44% (95% CI, 35–55%), whereas for ICI combined with chemotherapy and ICI combined with radiotherapy, the value was 80% (95% CI, 31–97%) and 97% (95% CI, 94–98%), respectively. No substantial heterogeneity was found in any of the three subgroups. Intracranial DCR was significantly higher when using ICI combined with radiotherapy than when using ICI monotherapy (OR [95% CI], 1.81 [1.53–2.14); *p* < 0.01) but not different when using ICI combined with chemotherapy (OR [95% CI], 1.48 [0.73–3.00]; *p* = 0.23).

Six [7,8,9,10,11,12], one [25], and two [13,14] studies reported intracranial CR rates when using ICI monotherapy, ICI combined with chemotherapy, and ICI combined with radiotherapy, respectively. Overall intracranial CR was 19% (95% CI, 9–36%), with substantial heterogeneity (*I*^2^ = 71%; *p* < 0.01) (see Supplementary Figure S1). Pooled intracranial CR, based on random-effects modeling, was 10% (95% CI, 4–22%), 20% (95% CI, 3–69%), and 46% (95% CI, 40–51%) when using ICI monotherapy, ICI combined with chemotherapy, and ICI combined with radiotherapy, respectively. No substantial heterogeneity was found in any of the three subgroups. Intracranial CR was significantly higher when using ICI combined with radiotherapy than when using ICI monotherapy (OR [95% CI], 1.58 [1.46–1.71]; *p* < 0.01).

### 3.5. Safety

**Figure 3:**
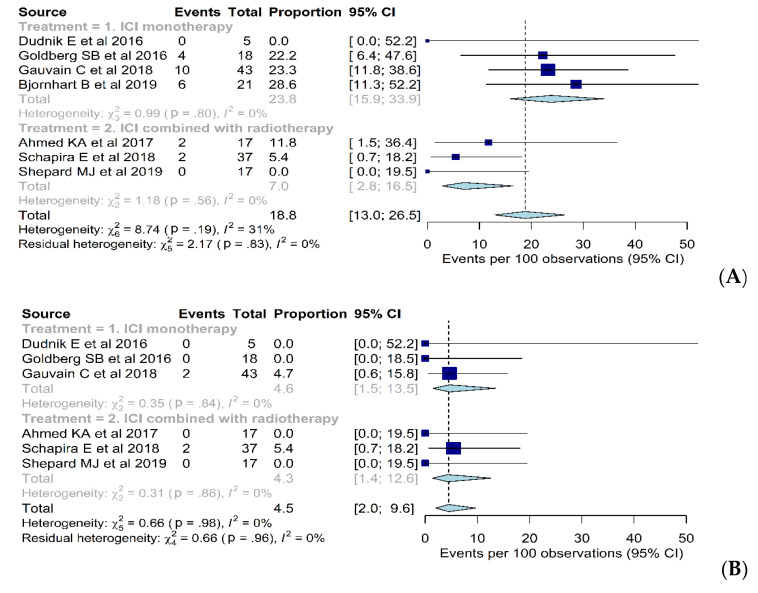
Forest plot of the (**A**) grade 3 or 4 adverse event rates and (**B**) grade 3 or 4 CNS-related adverse event rates when using immune checkpoint inhibitor (ICI) monotherapy, ICI combined with chemotherapy, and ICI combined with radiotherapy.

The pooled adverse event rates are summarized in Supplementary Table S1 and details of adverse events are described in Supplementary Table S2. Four [7,8,9,10] and three studies [13,26,27] reported grade 3 or 4 adverse event rates when using ICI monotherapy and ICI combined with radiotherapy, respectively. Overall grade 3 or 4 adverse event rates were 19% (95% CI, 13–27%) without substantial heterogeneity (*I*^2^ = 31%; *p* = 0.19) (Figure 3). The pooled grade 3 or 4 adverse event rates were 24% (95% CI, 16–34%) and 7% (95% CI, 3–17%) when using ICI monotherapy and ICI combined with radiotherapy, respectively.

Three [8,9,10] and three studies [13,26,27] reported grade 3 or 4 CNS-related adverse event rates when using ICI monotherapy and ICI combined with radiotherapy, respectively. Overall grade 3 or 4 CNS-related adverse event rates were 5% (95% CI, 2–10%), without a heterogeneity (*I*^2^ = 0%; *p* = 0.98) (Figure 3). The pooled grade 3 or 4 CNS-related adverse event rates were 5% (95% CI, 2–14%) and 4% (95% CI, 1–13%) when using ICI monotherapy and ICI combined with radiotherapy, respectively. Grade 3 or 4 CNS-related adverse event rates were not significantly different between the two arms (*p* = 0.93).

## 4. Discussion

In this meta-analysis, ICI treatment showed good intracranial responses across the treatment arms (overall ORR, 51%; DCR, 69%; CR, 19%). Although the number of included papers was small, ICI combined with radiotherapy (pooled ORR, 95%; DCR, 97%; CR, 46%) showed more promising results than ICI monotherapy (pooled ORR, 24%; DCR, 44%; CR, 10%). Regarding safety, the grade 3 or 4 adverse event rate was lower when using ICI combined with radiotherapy (7%) than when using ICI monotherapy (24%), and there was no significant difference in grade 3 or 4 CNS-related adverse event rate between the two groups (5% in ICI monotherapy, 4% in ICI combined with radiotherapy). Overall, these results indicate that ICI combined with radiotherapy seems to be a promising option for the treatment of NSCLC brain metastases. However, prospective trials are necessary to confirm this.

Radiation therapy acts on tumor via cytotoxicity and systemic pro-inflammatory effect which lead to activate host anti-tumor immune response which is enhanced by ICI treatment [28]. Therefore, it was hypothesized that and ICI treatment could have a synergistic effect, so-called abscopal effect, with an increase of drug efficacy. In a recent study for advanced NSCLC, pembrolizumab showed a better therapeutic response in patients who received previous radiation therapy than in those who did not [15] and in NCT02492568 trial, pembrolizumab after radiotherapy improved ORR at 12 weeks from 20% of pembrolizumab alone to 50% [29]. In the same context, our study showed that the patients who had combination therapy with ICI and radiation showed better intracranial response rate compared to ICI monotherapy, which can be explained by increased cytotoxicity and permeability of the blood-brain barrier induced by radiation therapy [30]. Several studies have been suggested that combining immunotherapy with radiotherapy can increase immune response [15,29], and many ongoing trials (NCT03168464, NCT03044626, NCT03391869) are investigating more detailed information about sequence of treatment arms, optimal dose and fractionations with different combinations of ICIs and radiotherapy. These further studies could provide an opportunity to establish new treatment strategies of NSCLC in specific situations (e.g., alternative option of surgery in early stage NSCLC, or neoadjuvant therapy before surgery).

Regarding safety, the pooled CNS-related grade 3 or 4 adverse event rate was similar between the two groups. Treatment-related necrosis, a representative CNS-related adverse event, has a significant effect on quality of life due to the accompanying focal neurologic deficits. A previous study showed that immunotherapy increases treatment-related necrosis in melanoma cases [31], especially when using ipilimumab, a drug targeting CTLA-4 [32]. However, in our meta-analysis study, subjects were patients with NSCLC, and the ICI combined with radiotherapy group used drugs targeting PD-1/PD-L1. Thus, pooled CNS-related grade 3 or 4 adverse events may have had no significant difference between the two treatment arms. This is in line with an existing study [33] that showed that the safety profile of PD-1/PD-L1 immunotherapy for NSCLC patients is acceptable.

With the introduction of immunotherapy, a growing number of patients experience pseudoprogression. Pseudoprogression occurs when an image initially looks like progression, but then a durable response is shown on following imaging. According to previous studies, the rate of pseudoprogression in NSCLC patients receiving immunotherapy varies from 0% to 6% [34,35,36,37,38,39,40]. RANO-BM and immuno-response evaluation criterion in solid tumors (iRECIST) are representative assessment criteria considering pseudoprogression. Unlike iRECIST, which is mainly used for solid tumors of the whole body, RANO-BM was developed for assessing the therapeutic response of brain metastasis only. The acceptance of RANO-BM for the evaluation of therapeutic responses to ICI and SRS has been recently increasing. As immunotherapy targeted approaches for brain metastasis increase, these new criteria will have more important meanings.

This meta-analysis has some limitations. First, the total number of included studies was rather small, and because each study did not report on both efficacy and safety indicators, in some groups, the number of included studies was even more insufficient. For example, only one study was included in the ICI combined with chemotherapy group [25]; so, there was no statistically significant result compared to that obtained from studies included in the ICI monotherapy group. In addition, the number of studies was insufficient to perform subgroup analysis. Second, with the exception of one study, all others [10] were conducted retrospectively. Due to this, there is a possibility of recall bias. Therefore, several prospective trials are needed to investigate the outcomes of ICI combined with radiotherapy, especially focusing on its comparison with ICI monotherapy and ICI combined with chemotherapy. Third, response assessment criteria were different across the included studies, and only two studies [13,14] used RANO-BM, which is most suitable for assessing the effect of ICI on brain metastasis. Furthermore, some studies [8,11,12,25,27] did not report their response assessment time. Lastly, comparison of grade 3 or 4 adverse event rate between the two groups was not possible due to incomplete data from the included studies. Nevertheless, this meta-analysis provides important information and suggests the need for future studies.

## 5. Conclusions

In conclusion, although the number of included studies was small, ICI combined with radiotherapy showed better intracranial efficacy than ICI monotherapy for the treatment of NSCLC brain metastases. CNS-related grade 3 or 4 adverse event rate was not statistically different between the two groups. Several prospective trials are needed to compare the efficacy of ICI combined with radiotherapy with that of ICI monotherapy.

## Figures and Tables

**Table 1:** Characteristics of 12 included studies.

Study (Publication Year)	Nation	Multicenter	Study Design	Recruitment Period	Response Criteria	Response Assessment Time after Initiation of Therapy	ICI Used	Radio-Therapy	Symptoms	Analysis	Treated No.	ICI - Line of Therapy			
												1st	2nd	3rd	3rd>
**ICI monotherapy**															
Bjornhart B et al (2019) [7]	Denmark	No	Retrospective	2015.09–2018.04	RECIST 1.1	8~9 weeks	Pembrolizumab or nivolumab	-	Mixed	Per-patient	21	2 *	7 *	7 *	5 *
Dudnik E et al (2016) [8]	Israel	Yes	Retrospective	2015.02–2015.12	mRECIST 1.1	NR	Nivolumab	-	Asymptomatic	Per-patient	5	-			
Gauvain C et al (2018) [9]	France	Yes	Retrospective	2015.05–2016.08	RECIST 1.1	2 months	Nivolumab	-	NR	Per-patient	30	-			
Goldberg SB et al (2016) [10]	USA	No	Phase II trial	2014.03–2015.05	mRECIST 1.1	8 weeks	Pembrolizumab	-	Asymptomatic	Per-patient	18	5 *	6 *	3 *	4 *
Hendriks LEL et al (2019) [24]	France, Netherlands	Yes	Retrospective	2012.11–2018.07	NR	6–9 weeks	Nivolumab	-	Mixed	Per-patient	5	0	1	2	2
Kim R et al (2019) [11]	Korea	Yes	Retrospective	2014.02–2016.11	mRECIST 1.1	NR	Pembrolizumab or nivolumab	-	NR	Per-patient	18	0 *	6 *	7 *	5 *
Song P et al (2019) [12]	China	No	Retrospective	2015.08–2018.02	RECIST 1.1	NR	Pembrolizumab or nivolumab or atezolizumab	-	Mixed	Per-patient	5	-			
**ICI combined with chemotherapy**															
Afzal MZ et al (2018) [25]	USA	No	Retrospective	2016.01–2017.12	RECIST 1.1	NR	Pembrolizumab	-	NR	Per-patient	5	3 **	3 **		
**ICI combined with radiotherapy**															
Ahmed KA et al (2017) [26]	USA	No	Retrospective	2014.02–2016.10	iRANO	2–3 months	Nivolumab or durvalumab	SRS or FSRT	NR	Per-lesion	49	-			
Schapira E et al (2018) [27]	USA	No	Retrospective	2012.01–2017.12	NR	NR	Pembrolizumab or nivolumab or atezolizumab	SRS	NR	Per-lesion	85	0	-		
Shepard MJ et al (2019) [13]	USA	No	Retrospective	2012.01–2018.12	RANO-BM	2–3 months	Pembrolizumab or nivolumab or atezolizumab	SRS	NR	Per-patient	16	-			
Singh C et al (2019) [14]	USA	No	Retrospective	2013.01–2016.12	RANO-BM	1.5, 3, 6, 9, and 12 months	Pembrolizumab or nivolumab or nivolumab + ipilimumab or atezolizumab	SRS	NR	Per-lesion	291	0	-		

ICI = immune checkpoint inhibitor; NR = not reported; RECIST = Response Evaluation Criteria in Solid Tumors; mRECIST = modified RECIST; RANO = Response Assessment in Neuro-Oncology; iRANO = immunotherapy RANO; RANO-BM = RANO brain metastases; SRS = stereotactic radiosurgery; FSRT = fractionated stereotactic radiotherapy. * Only previous systemic therapies were considered when determining the line of therapy. ** One patient was not radiographically evaluated.

**Table 2:** Summary of quality assessment according to the GRADE system.

Study (Publication Year)	Initial Rating	Risk of Bias	Imprecision	Inconsistency	Indirectness	Publication Bias	Large Magnitude of Effect	Quality of Evidence
**ICI monotherapy**								
Bjornhart B et al (2019) [7]	Low	Not serious	Not serious	Not serious	Not serious	Not serious	Not large	Low
Dudnik E et al (2016) [8]	Low	Not serious	Serious	Not serious	Not serious	Not serious	Not large	Very low
Gauvain C et al (2018) [9]	Low	Not serious	Not serious	Not serious	Not serious	Not serious	Not large	Low
Goldberg SB et al (2016) [10]	High	Not serious	Not serious	Not serious	Not serious	Not serious	Not large	High
Hendriks LEL et al (2019) [24]	Low	Not serious	Serious	Not serious	Serious	Not serious	Not large	Very low
Kim R et al (2019) [11]	Low	Not serious	Not serious	Serious	Not serious	Not serious	Not large	Very low
Song P et al (2019) [12]	Low	Not serious	Serious	Not serious	Not serious	Not serious	Not large	Very low
**ICI combined with chemotherapy**								
Afzal MZ et al (2018) [25]	Low	Not serious	Serious	Not serious	Not serious	Not serious	Not large	Very low
**ICI combined with radiotherapy**								
Ahmed KA et al (2017) [26]	Low	Serious	Not serious	Not serious	Not serious	Not serious	Not large	Very low
Schapira E et al (2018) [27]	Low	Serious	Not serious	Not serious	Not serious	Not serious	Not large	Very low
Shepard MJ et al (2019) [13]	Low	Not serious	Not serious	Not serious	Not serious	Not serious	Not large	Low
Singh C et al (2019) [14]	Low	Serious	Not serious	Not serious	Not serious	Not serious	Large	Low

**Table 3:** Pooled analysis of the included studies evaluating efficacy (random-effects model).

Treatment Arm	Intracranial ORR			Intracranial DCR			Intracranial CR		
	Proportion	OR (95% CI)	*p*-Value	Proportion	OR (95% CI)	*p*-Value	Proportion	OR (95% CI)	*p*-Value
ICI monotherapy	24 (14–39)	REF		44 (35–55)	REF		10 (4–22)	REF	
ICI combined with chemotherapy	80 (31–97)	1.90 (0.76–4.79)	0.14	80 (31–97)	1.48 (0.73–3.00)	0.23	20 (3–69)	1.22 (0.75–2.00)	0.36
ICI combined with radiotherapy	95 (92–97)	2.32 (1.96–2.75)	<0.01	97 (94–98)	1.81 (1.53–2.14)	<0.01	46 (40–51)	1.58 (1.46–1.71)	<0.01
Total	51 (17–84)	-	-	69 (42–88)	-	-	19 (9–36)	-	-

Values are expressed as proportion (95% confidence interval). ICI = immune checkpoint inhibitor; ORR = objective response rate (proportion of the patients who were confirmed as CR or PR); DCR = disease control rate (proportion of the patients who were confirmed as CR, PR, or SD); OR = odds ratio; REF = reference category.

## Supplementary Materials

The following are available online at https://www.mdpi.com/2075-4418/10/12/1098/s1, Figure S1. Forest plot of the intracranial complete response rates when using immune checkpoint inhibitor (ICI) monotherapy, ICI combined with chemotherapy, and ICI combined with radiotherapy; Table S1. Pooled analysis of the included studies evaluating safety (fixed-effect model); Table S2. Summary of grade 3 or 4 adverse events.
